# miR-7 is recruited to the high molecular weight RNA-induced silencing complex in CD8^+^ T cells upon activation and suppresses IL-2 signaling

**DOI:** 10.1261/rna.079030.121

**Published:** 2024-01

**Authors:** Matilda Toivakka, Katrina Gordon, Sujai Kumar, José Roberto Bermudez-Barrientos, Cei Abreu-Goodger, Rose Zamoyska, Amy H. Buck

**Affiliations:** 1 Institute of Immunology and Infection Research, School of Biological Sciences, University of Edinburgh, Edinburgh EH9 3FL, United Kingdom; 2 Institute of Ecology & Evolution, School of Biological Sciences, University of Edinburgh, Edinburgh EH9 3FL, United Kingdom

**Keywords:** CD8^+^ T cell, high molecular weight RISC, IL-2, miR-7, microRNA

## Abstract

Increasing evidence suggests mammalian Argonaute (Ago) proteins partition into distinct complexes within cells, but there is still little biochemical or functional understanding of the miRNAs differentially associated with these complexes. In naïve T cells, Ago2 is found almost exclusively in low molecular weight (LMW) complexes which are associated with miRNAs but not their target mRNAs. Upon T-cell activation, a proportion of these Ago2 complexes move into a newly formed high molecular weight (HMW) RNA-induced silencing complex (RISC), which is characterized by the presence of the GW182 protein that mediates translational repression. Here, we demonstrate distinct partitioning of miRNAs and isomiRs in LMW versus HMW RISCs upon antigen-mediated activation of CD8^+^ T cells. We identify miR-7 as highly enriched in HMW RISC and demonstrate that miR-7 inhibition leads to increased production of IL-2 and up-regulation of the IL-2 receptor, the transferrin receptor, CD71 and the amino acid transporter, CD98. Our data support a model where recruitment of miR-7 to HMW RISC restrains IL-2 signaling and the metabolic processes regulated by IL-2.

## INTRODUCTION

miRNAs are ∼22 nt small noncoding RNAs that bind to Argonaute (Ago) proteins within RNA-induced silencing complexes (RISC). The canonical function of the miRNA is to then direct RISC to messenger RNA targets, leading to suppression of translation and increased de-adenylation. The abundance of a miRNA has historically been considered the most important indicator of its functional potential inside the cell. However, many recent observations across plant and animal systems do not fit this dogma and suggest that miRNAs can exist in distinct RISCs with different functional properties (Olejniczak et al. 2013; Wu et al. 2013; Flores et al. 2014; La Rocca et al. 2015; Dalmadi et al. 2019). In particular, RISCs can exist in low molecular weight (LMW) or high molecular weight (HMW) complexes depending on the interaction partners of the Ago proteins (Hock et al. 2007; Landthaler et al. 2008; Olejniczak et al. 2013). HMW RISCs have been shown to bind the trinucleotide repeat containing 6 (TNRC6) family of proteins including GW182 (TNRC6A), which recruits proteins that mediate translational repression and degradation of the target mRNA (Behm-Ansmant et al. 2006; Eulalio et al. 2008; Lazzaretti et al. 2009; Lian et al. 2009; Fabian and Sonenberg 2012). Rather little is understood regarding the function of LMW RISCs which dominate in most cell lines and resting cells, including naïve T cells (La Rocca et al. 2015).

Upon stimulation of T cells by mitogenic signaling (Olejniczak et al. 2013) or T-cell receptor (TCR) signaling (La Rocca et al. 2015), the GW182 protein is induced and interacts with Ago proteins to promote the formation of HMW RISC. Here, we characterize the miRNA composition of HMW RISC and LMW RISC and identify functions of miRNAs enriched in HMW RISCs in CD8^+^ T cells from OT-I TCR transgenic mice that were stimulated with their physiological peptide ligands. Comparison of HMW and LMW RISCs revealed distinct differences in the miRNA partitioning between the complexes and showed enrichment of canonical reference length miRNAs (compared to isomiRs) in HMW RISC compared to LMW RISC. The data are consistent with a model where target association drives partitioning into HMW RISC. We identified miR-7 as a key HMW-enriched miRNA in CD8^+^ T cells and show, using synthetic miRNA inhibitors, that miR-7 modulates the production of Interleukin-2 (IL-2) and proteins downstream from IL-2 that are involved in T-cell growth and nutrient uptake.

## RESULTS AND DISCUSSION

### Formation of HMW RISC in CD8^+^ T cells upon antigen stimulation

**FIGURE 1.**
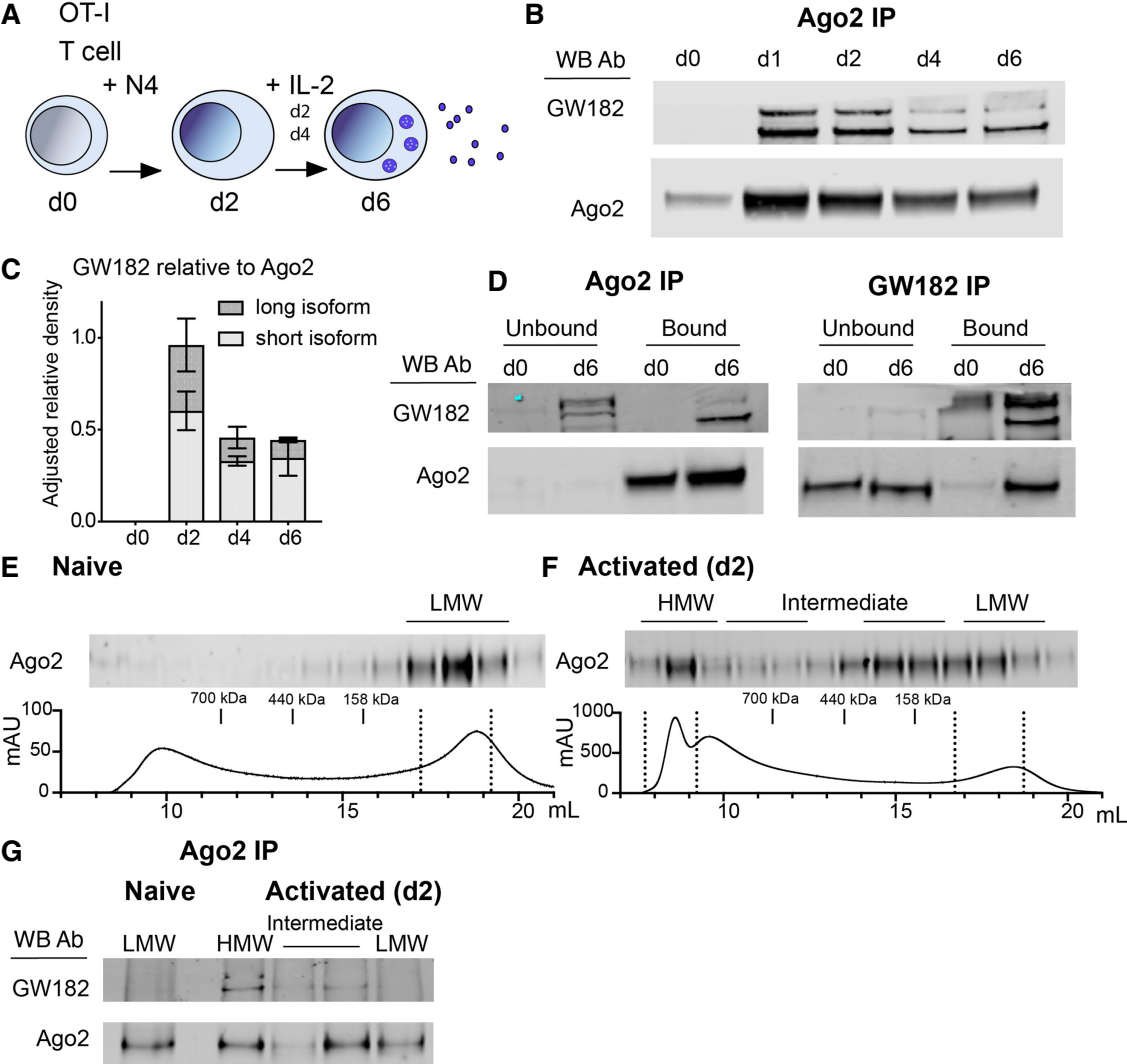
Ago2 forms HMW RISC with GW182 in activated CD8^+^ T cells. (*A*) Schematic of the experimental protocol indicating that T cells grow upon activation and develop into mature d6 cytolytic T cells capable of secreting effector molecules. Cytotoxic effector molecules are packaged in the endosome in the cytoplasm and then secreted, as indicated by the dense blue vesicles. (*B*) Ago2 was immunoprecipitated from cell lysates (each lane representing protein from 2 × 10^7^ cells), collected from a time-course of CD8^+^ T-cell activation, and western blots were probed with Ago2 and GW182 antibodies. (*C*) Quantification of the western blots measured on a LI-COR imager as GW182 band intensity relative to Ago2 band intensity, shown normalized to expression of Ago2 on d2. Mean and range from two independent experiments. (*D*) Western blot from Ago2 and GW182 IPs showing coimmunoprecipitated (bound) and residual protein (unbound) fractions, from naïve d0 and d6 activated CD8^+^ T-cell lysates (2 × 10^7^ cells per IP), probed with Ago2 and GW182 antibodies, as indicated. (*E*,*F*) Size exclusion chromatography fractions from naïve d0 (*E*) and d2 activated (*F*) CD8^+^ T-cell lysates. A total of 0.9 mg protein lysate (from 9 × 10^7^ cells) was added for (*E*) and 1.6 mg (from 8 × 10^7^ cells) for (*F*). Protein elution curves show fraction volume (in mL) and absorption at 280 nm (in milli-Absorption Units) with location in elution profile of protein size standards noted. Western blot from precipitated protein from fractions probed with Ago2 antibody. (*G*) Ago2 IP from HMW, intermediate and LMW fractions pooled together as indicated on western blots and elution curves (d2). Western blot of IP samples probed with Ago2 and GW182 antibodies.

To investigate the formation of RISC during CD8^+^ T-cell activation, we utilized OT-I CD8^+^ T cells that express a transgenic T-cell receptor (TCR) and respond to the ovalbumin-derived agonist peptide SIINFEKL (N4) presented by MHC H-2D^b^ molecules. Antigen stimulation drives activation of naïve CD8^+^ T cells, promoting transcription and translation of many genes and proteins, including transient expression of the cytokine IL-2, which plays important roles in promoting proliferation and T-cell differentiation (Feau et al. 2011). To sustain proliferation and to generate differentiated cytotoxic lymphocytes (CTLs), additional exogenous IL-2 cytokine was added to the cultures on day 2 (d2) and d4 (Fig. 1A). As shown in Figure 1B, GW182 coimmunoprecipitated with Ago2 from d1 after T-cell activation, as expected (La Rocca et al. 2015), and the two proteins remained associated during 6 d of culture, with a slight decrease in the amount of GW182 coimmunoprecipitated relative to Ago2 protein after d2 (Fig. 1C). The total levels of GW182 and Ago2 in the input samples are shown in Supplemental Figure 1A. Two isoforms have been described previously for GW182 which differ in their amino terminus, with the larger isoform named TNGW1 containing five additional exons including a TNR Q-repeat domain (Li et al. 2008); both were capable of being detected by the GW182 Ab used here. The shorter isoform predominantly immunoprecipitated with Ago2 by d4 (Fig. 1C). Coimmunoprecipitations of Ago2 and GW182 were performed to examine the stoichiometry and specificity of these interactions (Fig. 1D). Ago2 was efficiently immunoprecipitated (bound fraction) from both naïve (d0) and activated (d6) T cells as little Ago2 protein was present in the residual unbound fraction. The majority of the short form GW182 protein coimmunoprecipitated with Ago2 in the bound fraction at d6, leaving the longer isoform (and some background bands, see Supplemental Fig. 1A–C) in the unbound lysate (Fig. 1D). The GW182 immunoprecipitates confirmed the specificity of the GW182 bands detected by western blot and showed there was very little association between the immunoprecipitated longer isoform (TNGW1) and Ago2 at d0, whereas at d6 ∼50% of the Ago2 protein was associated with immunoprecipitated GW182 (bound), while the remaining Ago2 was detected in the unbound residual lysate. Overall these data indicate that a substantial amount of the short isoform of GW182 is associated with Ago2 at d6, whereas the longer isoform that is present at d0 has little association with Ago2.

Size exclusion chromatography confirmed that the GW182-bound Ago2 corresponded to a HMW RISC as described previously (La Rocca et al. 2015). As expected, all the detectable Ago2 in naïve T cells (d0) was found in LMW fractions (Fig. 1E). In activated cells (d2) however, Ago2 was found in multiple fractions, which we define as low, intermediate and HMW (Fig. 1F). We immunoprecipitated Ago2 from pooled fractions corresponding to LMW RISC from naïve (d0) cells (Fig. 1E), while from activated cells (Fig. 1F) we used three pooled fractions: HMW, two pools of intermediate fractions and LMW RISC. As shown in Figure 1G, GW182 coimmunoprecipitated with Ago2 in the HMW fractions and possibly some intermediate fractions from activated cells, but not in the LMW fractions. These data confirm that Ago2 and GW182 form HMW RISC in activated CD8^+^ T cells, with a fraction of Ago2 remaining GW182-unbound in LMW RISC.

### Differential partitioning of reference miRNAs and isomiRs into LMW and HMW RISC

**FIGURE 2.**
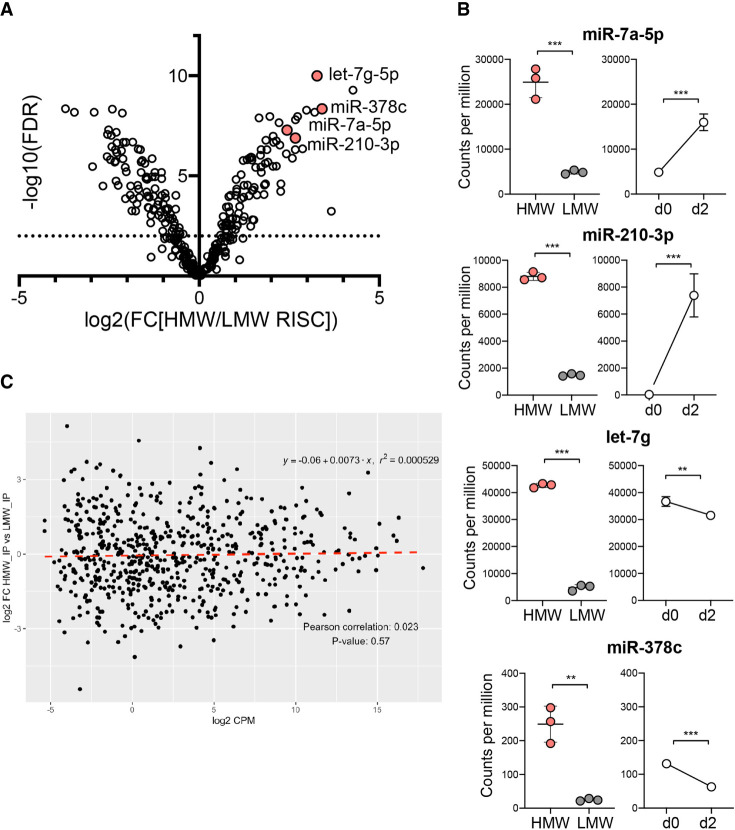
Specific miRNAs are found significantly enriched in HMW RISC in activated CD8^+^ T cells. (*A*) Differential expression of miRNAs between HMW and LMW RISC shown in a volcano plot of log_2_ fold change expression in HMW versus LMW RISC and −log_10_ of false discovery rate. Significant differences (FDR < 0.01) are *above* the dashed line. (*B*) Counts per million (CPM) of miRNAs in HMW and LMW RISC in d2 activated cells. Expression change is shown as CPM in Ago2 IP from naïve d0 and d2 activated cells. Data are from three biological replicates in one experiment. Statistical analysis was performed using a two-tailed unpaired student's *t*-test (*** *P* < 0.01, ** *P* < 0.05). (*C*) Partitioning of miRNAs in HMW RISC is not correlated with total expression levels of miRNAs based on Pearson correlation. *Y*-axis shows the log_2_ fold change of miRNA read counts in HMW IP versus LMW IP libraries and *X*-axis is the log_2_ counts per million (using the data described in *A* and *B*).

In order to compare the miRNA profiles of HMW RISC versus LMW RISC in activated CD8^+^ T cells, we then sequenced the small RNAs from immunoprecipitated Ago2 of each fraction (Supplemental Fig. 1D,E). The data were normalized to total read counts and the HMW and LMW RISC miRNAs were compared by differential expression analysis. We detected a total of 677 miRNAs of which 299 were analyzed (based on criteria of average counts per million [CPM] > 4). In this data set, 23 miRNAs were significantly enriched in HMW RISC with over fourfold enrichment (FDR < 0.01; Supplemental Table 1), and 28 were enriched in LMW RISC (Supplemental Table 2) as shown in Figure 2A. To determine the relationship between miRNAs induced upon CD8^+^ T-cell activation and miRNA partitioning in HMW and LMW RISC, cells were activated with peptide for 2 d. Activation of CD8^+^ T cells resulted in dramatic up- or down-regulation of specific miRNAs, both at the level of Ago2-bound miRNAs (Supplemental Fig. 2A) or total miRNA levels (Supplemental Fig. 2B,C), consistent with previous reports (Rodriguez-Galan et al. 2018). Of the miRNAs enriched in HMW RISC, miR-7 and miR-210 were strongly up-regulated, as shown by higher CPM in the Ago2 IP from activated T cells (d2) compared to Ago2 IP from naïve cells (d0), in contrast to miR-378c and let-7g that were down-regulated (Fig. 2B). The enrichment of specific miRNAs in HMW RISC is therefore not directly linked to their up- or de-regulation upon T-cell activation and is also not a consequence of overall abundance, with essentially no correlation between miRNA abundance and HMW RISC enrichment (Fig. 2C; Pearson correlation of 0.023 and *P*-value of 0.57). To further examine any potential specificity in partitioning of miRNAs into RISCs, we analyzed the composition of the miRNA reads in LMW versus HMW fractions, distinguishing the reference miRNA reference reads (as defined in miRBase) from isomiR reads that contain shorter or longer 5′ and/or 3′ ends that can impact miRNA stability and target recognition (Tomasello et al. 2021). We found a significantly larger proportion of reads corresponding to the reference miRNA in HMW fractions compared to LMW fractions (51.3% of reads compared to 39.3% of reads; Welch two sample *t*-test *P*-value = 8.5 × 10^−05^, Supplemental Fig. 3A), with over 60% of the reads in the LMW libraries corresponding to isomiRs.

Collectively our data are consistent with a model where the canonical functional activity of a miRNA is associated with HMW RISC, and this is likely to be defined by engagement with targets. Consistent with this, members of the same miRNA family (characterized by a shared seed sequence) displayed the same pattern in terms of enrichment in HMW versus LMW RISC (Supplemental Fig. 3B,C). For example, miR-17 family members, which regulate CD8^+^ T-cell proliferation and cell differentiation, were found up-regulated and enriched in HMW RISC (Supplemental Fig. 3C; Wu et al. 2012; Khan et al. 2013). Similarly, miR-449a and miR-449c that share a seed site with miR-34a, were up-regulated and enriched in HMW RISC (Supplemental Fig. 3C). Members of the let-7 family were down-regulated overall upon T-cell activation but significantly enriched in HMW RISC (Supplemental Fig. 3C), in agreement with previous results (La Rocca et al. 2015). Since the seed sites of the miRNAs are expected to define their targets, the commonalities among family members suggests that target expression in the activated cell could contribute to partitioning of the miRNAs to HMW RISC. Our data also reinforce the idea that the importance of a miRNA in canonical suppression of mRNA targets may have more to do with its association with HMW RISC than its overall abundance in the cell.

### miR-7 suppresses T-cell activation

Based on our data, we hypothesized that the miRNAs that are enriched in HMW RISC may be particularly important in regulating T-cell activation. As an example, we chose one of the miRNAs, miR-7, which was highly enriched in HMW RISC upon CD8^+^ T activation and was recently proposed to suppress CD4^+^ activation in liver injury (Zhao et al. 2021). The canonical biogenesis of miR-7 is subject to extensive regulation (Choudhury et al. 2013) and miR-7 is interesting as it has been shown to play a conserved role in regulating metabolism in mammalian tissues and fruit flies (Fernandez-de Frutos et al. 2019; Agbu et al. 2020). Given that naïve T cells undergo profound metabolic up-regulation upon activation (Buck et al. 2015), we hypothesized that miR-7 might also play a role in regulating T-cell metabolism. In cancer cells, miR-7 has been shown to be tumor-suppressive, and it targets components of the mammalian target of the rapamycin (mTOR) pathway (Fang et al. 2012; Wang et al. 2013; Zhao et al. 2015), a pathway essential for effector T-cell development (Powell et al. 2012). To determine the function miR-7 in CD8^+^ T cells, we used a locked nucleic acid (LNA) inhibitor that was delivered to the naïve T cells at the time of activation on d0. Using flow cytometry, we demonstrate efficient uptake of the fluorescently labeled miR-7 inhibitor on d1 and d2 post activation of the cells (Supplemental Fig. 4A). Trypsin treatment prior to flow cytometry reduced the signal for the surface coreceptor CD8β but caused only a slight decrease in the fluorescent signal from the inhibitors, consistent with their internalization (Supplemental Fig. 4B). Cell viability was not affected by the uptake of the miR-7 inhibitor or a control inhibitor (Supplemental Fig. 4C).

**FIGURE 3.**
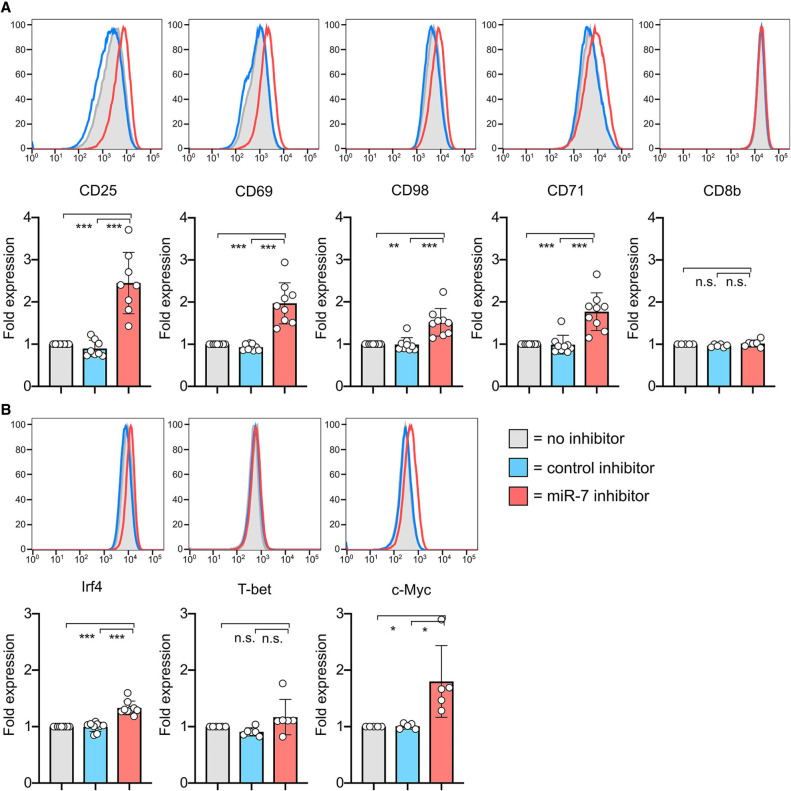
Inhibition of miR-7 during T-cell activation results in altered cell phenotype. (*A*,*B*) Cells were activated with SIINFEKL peptide in the presence of miR-7 inhibitor and expression of surface markers (*A*) and transcription factors (*B*) was measured on d2. Representative flow cytometry histograms are shown with graphs showing individual biological replicates pooled from 4–6 independent experiments, with “fold expression” calculated from the median fluorescence index and shown relative to “no inhibitor” control. Statistical analysis was performed using a one-sample *t*-test to compare “miR-7 inhibitor” to the hypothetical mean of 1 and a two-tailed unpaired student's *t*-test to compare “control inhibitor” and “miR-7 inhibitor” conditions.

We then examined whether the T-cell response to stimulation was altered by the inhibitors by measuring key parameters of activation, namely cell proliferation, cell size, expression of surface markers and of transcription factors by flow cytometry. Two days after activation, the cells had proliferated at a comparable rate (Supplemental Fig. 4D). Naïve T cells increase their mass upon activation proportionally to the strength of stimulation, which can be measured by increases in forward scatter (FSC) profiles by flow cytometry. On d2, N4 stimulated cells receiving the miR-7 inhibitor were found to be slightly larger, indicative of greater cellular mass (Supplemental Fig. 4E). We measured the expression of various receptors which are activated upon T-cell stimulation and regulate cell growth, such as the IL-2 receptor alpha chain, CD25; the surface activation marker, CD69; the transferrin receptor, CD71; and the heterodimeric large amino acid transporter SLC7A5/SLC3A2 (CD98) one and two days after activation in the absence or presence of the inhibitors. On d2, the cells receiving the miR-7 inhibitor specifically expressed higher levels of CD25, CD69, CD71, and CD98, whereas expression of a control surface receptor, CD8β, was unchanged (Fig. 3A). We also measured the expression of the transcription factors IRF4, T-BET, and c-MYC, that regulate CD8^+^ T-cell activation, metabolism and differentiation to effector cells. The cells receiving the miR-7 inhibitor consistently showed increased expression of c-MYC, and to a lesser extent IRF4, with no change in the expression of T-BET (Fig. 3B).

### miR-7 suppresses IL-2 signaling

Since inhibition of miR-7 had no effect on the activation phenotype of the T cells on d1 (Supplemental Fig. 4F) but showed strong phenotypic changes at d2, we hypothesized that miR-7 may be important in down-regulating the IL-2 pathway. Although the CD25 component of the high affinity IL-2R is induced by TCR stimulation initially, its expression is subsequently maintained by, and proportional to, the availability of IL-2 to the T-cell (Kim et al. 2006). Both TCR signals and IL-2 contribute to the up-regulation of CD98 that interacts with LAT1, and potentially CD69, to form the system L (“leucine preferring system”) amino acid transporter responsible for large neutral amino acid (LNAA) uptake in activated T cells (Sinclair et al. 2013; Cibrian et al. 2016). Key metabolic regulators such as the mTOR complex 1 (mTORC1) and c-MYC, that regulate the expression of glucose, glutamine and transferrin receptors, are also regulated by IL-2 signaling and system l-amino acid uptake (Preston et al. 2015; Rollings et al. 2018; Howden et al. 2019).

**FIGURE 4.**
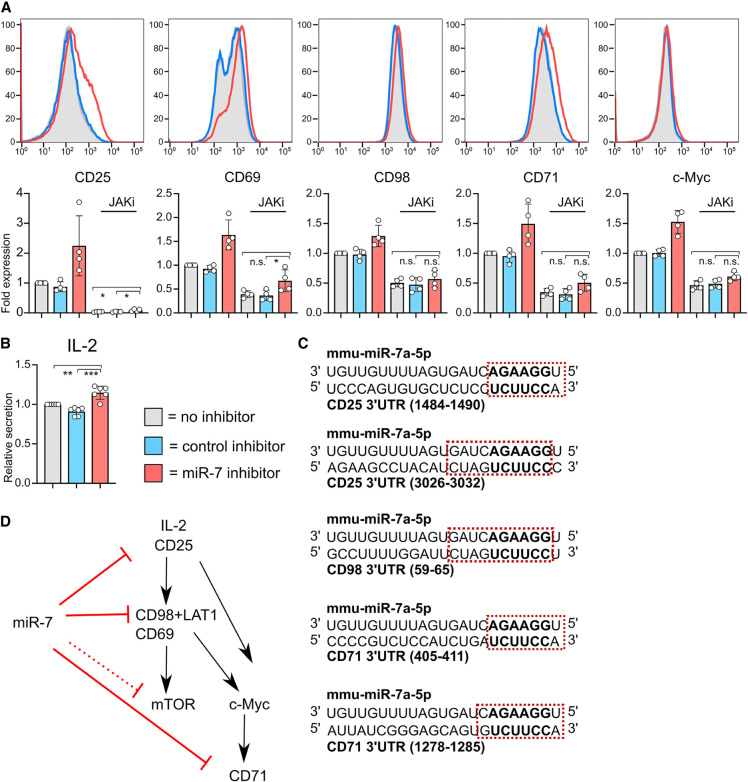
Blockade of JAK signaling partially abrogates miR-7 inhibition. (*A*) Cells were activated with SIINFEKL peptide ± miR-7 or control inhibitor. Additionally JAK inhibitor (tofacitinib) was added on d1. Expression of surface markers and transcription factors was measured on d2. Representative flow cytometry histograms are shown with pooled data from four independent experiments. Minus JAK inhibitor data are reprised from Figure 3. Fold expression calculated from the median fluorescence index is shown relative to “no miRNA inhibitor” control. Statistical analysis was performed using a one-way ANOVA with Tukey's multiple comparisons test, to compare the JAK inhibitor treated conditions. (*B*) IL-2 secreted on d1, measured by ELISA from culture supernatants. IL-2 receptor blocking antibody was included in cultures to prevent uptake and depletion of IL-2 by the T cells. The graph is showing six biological replicates from three independent experiments, as IL-2 secretion (measured in pg/mL from culture supernatant) relative to “no inhibitor” control. Statistical analysis was performed using a one-sample *t*-test to compare “miR-7 inhibitor” to the hypothetical mean of 1 and a two-tailed unpaired student's *t*-test to compare “control inhibitor” and “miR-7 inhibitor” conditions. (*C*) miR-7 target site location in 3′UTR of CD25, CD98, and CD71. A six-mer seed site is shown in bold and other complementary nucleotides are shown within the red square. (*D*) Schematic of proposed mechanism of action of miR-7 in CD8^+^ T cells.

To determine the effect of miR-7 on IL-2 signaling, we used a Janus kinase (JAK) inhibitor tofacitinib, which blocks JAK3 signaling downstream from the IL-2 receptor. The addition of the JAK inhibitor on d1 caused a complete down-regulation of CD25 and a strong reduction in the expression of CD69, CD71, and CD98 by d2, as expected since IL-2 is important in maintaining production of these proteins (Supplemental Fig. 4G; Rollings et al. 2018). Addition of the JAK inhibitor lessened the phenotypic differences between miR-7-inhibited and control cultures (Fig. 4A) and was consistent with a slight but statistically significant increase in IL-2 production which was observed in the presence of the miR-7 inhibitor (Fig. 4B). These data indicate that miR-7 dampens the production of IL-2 following TCR stimulation, which in turn modulates the expression of a number of molecules downstream from IL-2R signaling by d2 of activation. However, even following inhibition of JAK3, there was higher abundance of CD25, CD69, CD71, and to a lesser extent CD98 and c-MYC in the presence of the miR-7 inhibitor (Fig. 4A). We note that CD25, CD98, and CD71 are predicted miR-7 targets based on TargetScan (Fig. 4C), and CD98 and CD71 have been shown to be directly regulated by miR-7 in cancer cell lines (Nguyen et al. 2010; Miyazawa et al. 2018). While additional targets of miR-7 may also be important in T cells, our findings demonstrate that miR-7 regulates IL-2 signaling in CD8^+^ T cells on multiple levels, impacting IL-2 production, signaling through the high affinity IL-2R by targeting CD25, and modulating nutrient uptake by directly regulating transporters such as CD98 for amino acids and CD71 for transferrin (Fig. 4D). This could be important in conditions of persistent inflammation to avoid T-cell exhaustion (Wherry and Kurachi 2015; Gaud et al. 2018). Our work adds to diverse contexts in which miR-7 operates and supports an emerging role of this miRNA in inflammation and immune signaling.

Interestingly, in T cells GW182 expression and HMW RISC formation is at least partly regulated by mTOR signaling following T-cell activation (La Rocca et al. 2015) and IL-2 stimulates mTORC1 activity in T cells (Powell et al. 2012; Ross and Cantrell 2018). We propose therefore that the formation of HMW RISC following mTOR signaling is one feedback pathway to regulate T-cell activation through miR-7 and potentially other miRNAs. This type of feedback is likely to occur in various cell contexts since the formation of HMW RISC is regulated by mitogenic signals, and the deprivation of growth factors or glucose caused decreased expression of GW182 and formation of a LMW RISC in cell lines (Olejniczak et al. 2013).

In summary, we have confirmed the presence of LMW RISC in CD8^+^ T cells and the induction of HMW RISC when these are stimulated with antigenic peptide. We have shown enrichment of specific miRNAs and miRNA families in HMW RISC and demonstrated that one of these, miR-7, is a novel regulator of T-cell activation and metabolism that suppresses IL-2 signaling and nutrient uptake. There remain open questions in regard to the function, composition and recruitment of miRNAs to HMW RISC, but our data show that focusing on miRNAs enriched within HMW RISC can lead to the identification of new functionally relevant miRNAs that play important roles in T-cell activation and differentiation. This work further highlights the concept that miRNA abundance is not a direct correlate for miRNA functional activity.

## MATERIALS AND METHODS

### Materials availability

This study did not generate new unique reagents.

### Mice and primary cell cultures

All mice were maintained and bred in pathogen-free conditions at the University of Edinburgh animal facilities in accordance with the UK Home Office and local ethically approved guidelines. OT-I transgenic mice (Hogquist et al. 1994) on the Rag-1KO C57BL/6 genetic background were used, as well as OT-I mice containing a tagged Ago2 knock-in (Schmolka et al. 2018) for the experiments shown in Figures 1 and 2, and Supplemental Figures 1–3. Mice were used at 7–12 wk of age and animal sex was not expected to have a significant influence on outcomes. Single cell suspensions were prepared mechanically from mouse lymph nodes using a 70 µm filter. Cells were grown in Iscove's Modified Dulbecco's Medium (IMDM, Sigma Aldrich) supplemented with 10% heat inactivated fetal calf serum (FCS, Gibco), 100 u/mL streptomycin (Gibco), 100 µg/mL penicillin (Gibco), 2 mM l-glutamine (Gibco), and 50 µM β-mercaptoethanol. The cells were grown at 37°C.

### T-cell culture

To activate OT-I cells, 2 × 10^6^ cells/mL were grown in media supplemented with 10 nM N4 for 2 d. For effector cell differentiation, cells were activated with N4 as before for 2 d, then resuspended at 2 × 10^5^ cells/mL in media supplemented with 20 ng/mL IL-2 (PeproTech). miRNA inhibitors (miRCURY LNA miRNA power inhibitor with or without 5′-FAM, Qiagen) were added directly to culture media at the time of activation and were taken up by the cells by spontaneous translocation across the cell membrane. A total of 500 nM miRNA inhibitor (mmu-mir-7a-5p) or control inhibitor (negative control A) was added to naïve T cells in cell culture media with N4, and phenotype was assessed on d1 and d2. For JAK inhibition, tofacitinib (Stratech) was added to the cultures on d1 at a final concentration of 200 nM, and cell phenotype was measured on d2.

### Flow cytometry

To measure cell proliferation, cells were stained with a CellTrace Violet Cell Proliferation kit (Thermo Scientific) prior to culturing. Flow cytometry staining was undertaken in 96-well round-bottom plates with a minimum of 200,000 cells per well. To distinguish live cells, the cells were stained with a LIVE/DEAD Fixable Aqua Dead Cell Stain kit (Thermo Scientific). For surface staining, the cells were incubated in Facs buffer (2.5% FBS and 0.05% sodium azide in PBS) with labeled antibodies, all Thermo Scientific unless otherwise indicated: anti-CD8β, anti-CD25, anti-CD69 (BioLegend), anti-CD71 (BioLegend) and anti-CD98. Intranuclear staining was done using the eBioScience Foxp3/Transcription Factor Staining kit (Thermo Scientific) and anti-Irf4, anti-T-bet, and anti-c-Myc (Cell Signaling) antibodies. Flow cytometry was performed on a MacsQuant Analyzer 10. Flow cytometry data were analyzed using FlowJo 10 software. Flow cytometry data were generated within the Flow Cytometry and Cell Sorting Facility in Ashworth, King's Buildings at the University of Edinburgh.

### ELISA

To measure IL-2 production, cells were cultured in the presence of an anti-CD25 blocking antibody (2BScientific, PC61). IL-2 was measured from culture supernatants using a mouse IL-2 ELISA kit (Thermo Scientific).

### Western blotting

Cells were lysed in lysis buffer (50 mM Tris/HCl pH = 7.8, 300 mM NaCl, 1% Triton X100, 5 mM EDTA, 10% glycerol) containing protease inhibitors (cOmplete Protease Inhibitor Cocktail tablets, Roche), and if required, RNase inhibitors (RNasin Ribonuclease Inhibitor, Promega). Cell lysates were denatured in LDS sample buffer (Thermo Scientific) containing 10% β-mercaptoethanol at 95°C. Proteins were fractionated on 4%–12% Bis-Tris gels (NuPAGE, Thermo Scientific) and transferred onto PVDF membranes (Immobilon-FL, Merck) using the wet-transfer method. The membrane was incubated with an Ago2 antibody (a generous gift from Dónal O'Carrrol) or GW182 antibodies (Bethyl Laboratories, A302-329A), then secondary labeled anti-mouse IgG (IRDye 800CW, LI-COR) or anti-rabbit IgG (Alexa Fluor 680, Thermo Scientific) antibodies in Odyssey blocking buffer containing 0.1% Tween-20 (LI-COR). The membrane was scanned using the Odyssey Clx Imaging System (LI-COR). Western blot data were analyzed using ImageStudioLite (LI-COR).

### Immunoprecipitations

Immunoprecipitations were undertaken using magnetic Protein G Dynabeads (Thermo Scientific) conjugated with anti-Ago2 or anti-GW182 antibodies. Cell lysates were incubated with the beads overnight after which the unbound fraction was collected. The beads were then washed using the following conditions: 1× LS-IP wash (50 mM Tris/HCl pH = 7.5, 0.3 M NaCl, 5 mM MgCl_2_, 0.5% Triton x100, 2.5% glycerol), 2× HS-IP wash (50 mM Tris/HCl pH = 7.5, 0.8 M NaCl, 10 mM MgCl_2_, 0.5% Triton x100, 2.5% glycerol), 1× LS-IP wash, 1× PNK wash (50 mM Tris/HCl pH = 7.5, 10 mM MgCl_2_, 0.5% Triton X100, 50 mM NaCl). The IP-bound fraction was eluted by resuspending the beads in LDS sample buffer (Thermo Scientific) containing 10% β-mercaptoethanol and incubating at 70°C for 10 min with shaking.

### Size exclusion chromatography

Cells were flash-frozen in liquid nitrogen and lysed in 0.5 mL Superose 6 buffer (150 mM NaCl, 10 mM Tris/HCl pH = 7.5, 2.5 mM MgCl_2_, 0.01% reduced Triton x100, 1 mM DTT) for 20 min. The lysate was cleared by centrifugation at top speed and filtering through a 20 µm filter. Protein concentration was measured with a Qubit 3.0 fluorometer (Thermo Scientific) using the Qubit Protein Assay kit (Thermo Scientific). Size exclusion chromatography was performed at the Edinburgh Protein Production Facility. The sample was loaded on the Superose 6 column, washed with Superose 6 buffer (omitting DTT), and 0.5 mL fractions were collected. Protein was extracted from the fractions by trichloroacetic acid (TCA) precipitation.

### Reverse transcription and qPCR

RNA was extracted from cells in TRIzol reagent (Thermo Scientific) using the Direct-zol kit (Zymo Research). The RNA was reverse transcribed to cDNA using the miScript RT kit (Qiagen). miRNAs were quantified using miScript Primer Assays (Qiagen) and QuantiText SYBR Green PCR Master Mix (Qiagen) on a LightCycler 480 Instrument II (Roche). qPCR data were analyzed in Microsoft Excel. Data were first normalized to snRNA U6, then calculated as fold change to naïve, using the ΔΔCt value method.

### Small RNA library preparation

For small RNA library preparation, RNA was isolated from Ago2-IP samples, as well as the IP input and unbound samples. The samples were resuspended in QIAzol lysis reagent, and RNA was extracted using the miRNeasy kit (Qiagen) and eluted in 100 µL dH_2_O. The RNA was then ethanol precipitated by adding 2.5× volume 100% ethanol, 30 µL sodium acetate and 1 µL GlycoBlue Coprecipitant (Thermo Scientific) to the samples which were then incubated overnight at −20°C. The following day, the samples were centrifuged at full speed for 30 min, then washed twice with 70% ethanol. The pellets were air-dried on ice, then resuspended in 10–15 µL dH_2_O and quantified with a Qubit 3.0 fluorometer (Thermo Scientific) using the Qubit RNA HS Assay kit (Thermo Scientific). Small RNA libraries were prepared using the CleanTag Small RNA Library Preparation kit (TriLink) using manufacturer's instructions and 21 cycles. The libraries were size selected to include 145–160 bp and the input, IP-unbound and IP-bound libraries were pooled at a 1:1:2 ratio. The samples were sequenced using NovaSeq 50 bp paired-end sequencing at Edinburgh Genomics.

### miRNA and isomiR data analysis

RNA sequencing data were processed to create miRNA count files. To assess the presence and counts of individual miRNAs, we used the miRNA counting tool QuickMIRSeq (Zhao et al. 2017). QuickMIRSeq does its own read trimming and length selection using cutadapt, which we set to trim the 3′ adapter TGGAATTCTCGGGTGCCAAGG, the 5′ adapter AGATCGGAAGAGCACACGTCT, and choose only reads between 18 and 28 bp in length for assigning to known miRNAs. The default QuickMIRSeq settings were used for identifying mouse miRNAs. The QuickMIRSeq output file miR.filter.Counts.csv was used for all downstream analyses. The miRNA count files were then uploaded on the Degust (version 3.1.0) (Powell 2015) web tool for visualization of differential expression analysis. The differential analysis was performed using the Voom/Limma method and visualized on Degust. For the isomiR anlaysis we used miraligner (Pantano et al. 2010) on mouse miRNA hairpin sequences taken from miRBase v22.1 (Kozomara et al. 2019). Miraligner parameters are the following: allow 1 mismatch, trim up to 3 nt from the ends, allow up to 3 nt as nontemplate additions. The resulting tables were processed with isomiRs R package version 1.26.0 (10.18129/B9.bioc.isomiRs). The files were imported with the IsomirDataSeqFromFiles function setting the canonicalAdd parameter as true. After reading the files, the read counts were aggregated according to the categories reference or isomiR with the help of the dplyr v1.1.2 package (https://github.com/tidyverse/dplyr), and the plots were made with ggplot 2 v3.4.2 (https://ggplot2.tidyverse.org). Statistical significance was calculated with a Welch two sample *t*-test comparing reference and isomiRs percentage in HMW versus LMW.

### Statistical analysis

Statistical analysis was performed on a GraphPad Prism unless noted otherwise. Details of statistical tests used and number of biological and/or technical replicates can be found in the figure legends.

## DATA DEPOSITION

The small RNA sequencing data are available at the GEO repository under accession number GSE151555.

## SUPPLEMENTAL MATERIAL

Supplemental material is available for this article.
